# DNA copy number concentration measured by digital and droplet digital quantitative PCR using certified reference materials

**DOI:** 10.1007/s00216-015-8458-z

**Published:** 2015-01-20

**Authors:** Philippe Corbisier, Leonardo Pinheiro, Stéphane Mazoua, Anne-Marie Kortekaas, Pui Yan Jenny Chung, Tsvetelina Gerganova, Gert Roebben, Hendrik Emons, Kerry Emslie

**Affiliations:** 1 Institute for Reference Materials and Measurements, Joint Research Centre, European Commission, Retieseweg 111, 2440 Geel, Belgium; 2 Bioanalysis Group, National Measurement Institute, 1 Suakin Street, Pymble, New South Wales 2066 Australia

**Keywords:** Digital PCR, Droplet digital PCR, Reference materials, Optical microscopy, Areal equivalent diameter

## Abstract

The value assignment for properties of six certified reference materials (ERM-AD623a–f), each containing a plasmid DNA solution ranging from 1 million to 10 copies per μL, by using digital PCR (dPCR) with the BioMark™ HD System (Fluidigm) has been verified by applying droplet digital PCR (ddPCR) using the QX100 system (Bio-Rad). One of the critical factors in the measurement of copy number concentrations by digital PCR is the partition volume. Therefore, we determined the average droplet volume by optical microscopy, revealing an average droplet volume that is 8 % smaller than the droplet volume used as the defined parameter in the QuantaSoft software version 1.3.2.0 (Bio-Rad) to calculate the copy number concentration. This observation explains why copy number concentrations estimated with ddPCR and using an average droplet volume predefined in the QuantaSoft software were systematically lower than those measured by dPCR, creating a significant bias between the values obtained by these two techniques. The difference was not significant anymore when the measured droplet volume of 0.834 nL was used to estimate copy number concentrations. A new version of QuantaSoft software (version 1.6.6.0320), which has since been released with Bio-Rad’s new QX200 systems and QX100 upgrades, uses a droplet volume of 0.85 nL as a defined parameter to calculate copy number concentration.

**Graphical Abstract:**
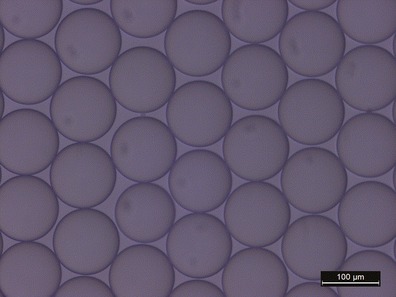
Monolayer of droplets generated by the droplet generator and observed under an optical microscope

Monolayer of droplets generated by the droplet generator and observed under an optical microscope

## Introduction

Quantification of nucleic acid molecules by quantitative PCR (qPCR) has been developed during the last 25 years in many applications of green and red biotechnologies and has progressively reached a level of maturity [1]. qPCR has been applied to gene expression quantification [2, 3], forensic DNA quantification [4, 5] and clinical [6, 7] and veterinary [8] virology diagnostics, all reviewed recently.

Quantification of nucleic acids by qPCR is commonly achieved by using a calibration curve, constructed by measuring the number of cycles required for the fluorescent signal to reach a threshold level for known amounts of target DNA in a parallel set of reactions. The threshold fluorescent level is calculated from the initial cycles, and this cycle number (Cq value) is proportional to the number of copies of template in the sample. The use of calibration curves has basically two consequences.

First, it requires that the exact quantity value carried by a calibration material (measurement standard) is determined by independent means using, e.g. spectrophotometry or an intercalating dye. Spectrophotometric methods rely on the molar absorptivity (or the molar absorption coefficient) of pure nucleic acid solutions used in the equation based on the Lambert-Beer law [9]. The molar absorptivity depends upon the chemical nature (deoxyribonucleic or ribonucleic) and conformation (single or double stranded) of the nucleic acid molecule as well as its degree of purity. The fraction of the absorbed light will depend on how many molecules the light interacts with, being the molecule of interest but also contaminants that have been co-extracted. Despite those limitations, spectrophotometric methods are widely used to quantify nucleic acids in solutions. Fluorometric methods using intercalating dyes have as a major advantage their higher sensitivity compared to spectrophotometric methods, allowing a detection down to 25 pg/mL dsDNA [10]. However, these measurements need to be calibrated using the quantity value of a material having ideally the same chemical nature and molecular weight as the analyte of interest. This circular problem cannot be solved without the use of a reference material that is certified for its nucleic acid concentration, a reason why several metrology institutes are developing such materials.

The use of an external measurement standard being an amplicon [11], cut or uncut plasmid DNA [12, 13], cosmid [14] or complementary DNA [15] to calibrate qPCR has a second drawback. A number of strategies have been used to prepare and apply a standard with similar properties to the target DNA. These include treating genomic DNA with a cocktail of restriction enzymes, DNA ultrasonication [16], shredding methods [17] or using a Bayesian approach that takes into account several sources of uncertainty [18]. A few studies have shown that those standards could behave in a similar way as the nucleic acid molecules that were targeted [19, 20]. However, factors such as DNA stability, base composition, secondary structure and presence of complex mixtures of non-target DNA can significantly alter the PCR amplification performance, making the DNA quantification dependent and only traceable to the calibrants that have been used [21].

Digital PCR (dPCR) technology [22] is an end-point measurement that overcomes the dependency on a DNA calibrant, and it has recently been used as an alternative method to assign copy number concentrations of defined nucleic acids in solution [23–26]. Some prerequisites of the technology have already been discussed [27], and recommendations were published on how to properly report digital PCR results [28]. The nucleic acid targets must be randomly distributed among the partitions, and the conditions of the PCR assays must be optimised to ensure that a single copy of the target is indeed amplified during the PCR. Since the calculation of the copy number concentration includes dividing the copy number estimate by the assay volume, knowing the correct partition or droplet volume is a key factor when measuring DNA concentrations using digital PCR [29]. The partition or droplet volumes as provided by the manufacturer are utilised by the majority of laboratories without further independent verification. The potential error on that volume is not important if a ratio between two concentrations of DNA targets is reported, but it can generate a significant bias when an absolute copy number concentration is measured.

In this study, we used a set of reference materials that has been certified by using dPCR for the absolute copy number concentration of a *BCR-ABL* target [24, 30] applying the BioMark™ HD System (Fluidigm). The certified concentrations were verified using results from the QX100 droplet digital PCR (ddPCR) system (Bio-Rad). In parallel, the average droplet volume was measured and the copy number concentration reported by the ddPCR QuantaSoft software (Bio-Rad) was corrected by taking into account the real average droplet volume.

## Materials and methods

### Test material

ERM-AD623a, ERM-AD623b, ERM-AD623c, ERM-AD623d, ERM-AD623e and ERM-AD623f (Joint Research Centre-Institute for Reference Materials and Measurements (JRC-IRMM)) were used as certified reference materials containing (1.08 × 10^6^ ± 0.13 × 10^6^), (1.08 × 10^5^ ± 0.11 × 10^5^), (1.03 × 10^4^ ± 0.10 × 10^4^), (1.02 × 10^3^ ± 0.09 × 10^3^), (1.04 × 10^2^ ± 0.10 × 10^2^) and (10.0 ± 1.5) copies (cp)/μL, respectively, of a double-stranded linearised plasmid [24]. The plasmid is in a 1-mmol/L Tris, 0.01-mmol/L EDTA pH 8.0 buffer (T_1_E_0.01_) supplemented with 50 mg/L of transfer RNA from *Escherichia coli* and used undiluted in the ddPCR experiment unless otherwise mentioned. The copy number concentrations and related uncertainties were established using the measurement data of three (National) Metrology Institutes each using their own dPCR apparatus from Fluidigm (i.e. the BioMark™ HD System) and two validated assays as described earlier [24].

### Digital PCR

Eppendorf DNA LoBind tubes (VWR International, cat no. 0030108051, Leuven, Belgium) as well as calibrated Rainin Pipet-Lite XLS+ single channel micropipettes (Mettler-Toledo S.A., Zaventem, Belgium) were used throughout this study. For the measurement of copy number concentrations of the six plasmid solutions by dPCR, 10 μL of ERM-AD623a, ERM-AD623b, ERM-AD623c and ERM-AD623d was gravimetrically diluted in T_1_E_0.01_ buffer to obtain DNA samples at a nominal concentration of 500 cp/μL. ERM-AD623e and ERM-AD623f were used undiluted in the dPCR assay. A volume of 19.7 μL of the DNA sample was further mixed with 30.3 μL of pre-sample mix solution, and 9 μL of this mixture was loaded on five panels of the 12.765 digital Array™ IFCs from Fluidigm (BIOKE, Leiden, Netherlands). The pre-sample mix solution contained the primers and probes for the *BCR-ABL* b3a2 transcript and for the *ABL* transcript (duplex PCR conditions) at final concentrations mentioned in Table 1 together with 20× GE sample loading reagent (Fluidigm) and TaqMan® Universal PCR MasterMix (Applied Biosystems, Ghent, Belgium) as recommended by the manufacturer. The PCR was performed according to the specifications listed in Table 2. The PCR runs were then analysed with the Fluidigm Digital PCR software version 3.0.2 using the following settings: quality threshold 0.4; linear baseline correction; automatic Ct threshold method; and target Ct range between 20 and 49. In case of low background noise, the results were analysed using a linear derivative baseline correction and a manual Ct threshold setting.

**Table 1:** Primers and probes used to amplify the *BCR-ABL* b3a2 transcript by dPCR and ddPCR

PCR assay	Primers and probe	Sequence	Final concentration [μM]	Amplicon size [bp]
*BCR-ABL* b3a2	F-primer	5′-TCCGCTGACCATCAAYAAGGA-3′	0.3	149
	R-primer	5′-CACTCAGACCCTGAGGCTCAA-3′	0.3	
	Probe	5′-(6-VIC)CCCTTCAGCGGCCAGTAGCATCTGA-(MGB)-3′	0.2	
*ABL*	F-primer	5′-TGGAGATAACACTCTAAGCATAACTAAAGGT-3′	0.3	122
	R-primer	5′-GATGTAGTTGCTTGGGACCCA-3′	0.3	
	Probe	5′-(6-FAM)CCATTTTTGGTTTGGGCTTCACACCATT-(TAMRA)-3′	0.2

**Table 2:** Thermal cycle protocol used for both dPCR and ddPCR protocols

Name	Phase	Time [s]	Temperature [°C]	Repeats
UNG and hot start	UNG	120	50	1
	Hot start	600	95	
PCR cycles	Denaturation	15	95	50
	Annealing	60	60	

### Droplet digital PCR

The copy number concentrations of the six plasmid solutions were measured by ddPCR using the QX100™ Droplet Digital™ PCR system (Bio-Rad, Temse, Belgium). A volume of 33.6 μL was pipetted from each CRM vial and mixed with 62.4 μL pre-sample solution. The pre-sample solution contained 48 μL of ddPCR Supermix (Bio-Rad, cat no. 186-3010), 4.8 μL forward and reverse primers and the probe for the *BCR-ABL* b3a2 transcript at the same final concentrations as those used for the dPCR experiments. ERM-AD623f, e and d were used undiluted and ERM-AD623c, b and a were gravimetrically diluted with T_1_E_0.01_ to a nominal concentration of 1000 cp/μL. Twenty microliters of this solution was pipetted in eight compartments of the Droplet Generator DG8 Cartridge (Bio-Rad, cat no. 186-3008) and droplets were generated. The entire droplet emulsion volume was further loaded in a semi-skirted and PCR-clean 96-well PCR plate (Eppendorf, Leuven, Belgium). The loaded 96-well PCR plate was then heat sealed with pierceable foil in the PX1™ PCR Plate Sealer and placed in a C1000 Touch™ Thermo Cycler (both from Bio-Rad). The same thermal cycling conditions were applied as those used for dPCR (described in Table 2) except that the UNG step was omitted and only 40 PCR cycles were run. After PCR amplification, the droplets were analysed in a QX100™ droplet reader (Bio-Rad), and the absolute quantification of PCR targets was analysed using QuantaSoft™ software version 1.3.2.0 with a threshold placed at an amplitude between 3000 and 4002.

The copy number concentration (*T*_c_) (in cp/μL) was also calculated using Eq. (1) taking into account the number of droplets analysed (*C*), the number of positive droplets (*H*), the average volume of a droplet determined experimentally (*V*_d_ in μL) and the final dilution factor of the sample (*D*_f_).

1 $$ {T}_{\mathrm{c}}={D}_{\mathrm{f}}\times \left(\frac{1}{C\times V\mathrm{d}}\right)\times \frac{\left( \log \left(1-\frac{H}{C}\right)\right)}{\left( \log \left(1-\frac{1}{C}\right)\right)} $$

The final dilution (*D*_f_) was calculated using Eq. (2) as the product of the dilution factor of the sample (*D*_s_) (if any) and the dilution factor of the DNA solution in the assay (*D*_a_).

2 $$ {D}_{\mathrm{f}}={D}_{\mathrm{s}}\times {D}_{\mathrm{a}} $$

The volume of each sample in the assay was determined by dividing the sample mass (*m*_DNA sol_) by the density of the solution (*δ*_DNA sol_). The density of the pre-sample mix (*δ*_p sol_) was determined by weighing 10 times 100 μL of the pre-sample mix (*m*_p sol_) on a ME235P analytical microbalance (Sartorius, Vilvoorde, Belgium). Each mass was divided by the pipetted volume to calculate the density. A density of 1.03532 g/mL for the pre-sample mix (*δ*_p sol_) and a density of 1.0000 g/mL for the sample (*δ*_DNA sol_) were taken into account in the calculation of the dilution factor in the assay (*D*_a_) (Eq. 3).

3 $$ {D}_{\mathrm{a}}=\frac{\frac{m_{\mathrm{DNA}\ \mathrm{sol}}}{\delta_{\mathrm{DNA}\ \mathrm{sol}}}+\frac{m_{\mathrm{p}\ \mathrm{sol}}}{\delta_{\mathrm{p}\ \mathrm{sol}}}}{\frac{m_{\mathrm{DNA}\ \mathrm{sol}}}{\delta_{\mathrm{DNA}\ \mathrm{sol}}}} $$

### Droplet volume sizing by optical microscopy

Average droplet volumes were determined for three DG8-186-3008 cartridges, each prepared on a different day. Droplet generation and acquisition of the optical microscopy images was performed on the same day. Image analysis was performed afterwards, off-line. For each of the three cartridges, a volume of 10 μL containing the droplets generated in the DG8-186-3008 cartridges was carefully pipetted into the chamber of an Ibidi plate (Ibidi μ-slide VI-flat non-coated, Proxylab sprl, Beloeil, Belgium). The Ibidi plates were held at an angle to allow the formation of a uniform monolayer of droplets for better imaging. Four wells were randomly selected from each of the three different droplet generator cartridges for this analysis. Between 140 and 160 droplets were measured in each selected well. Each cartridge was analysed on a different day providing measurement of 1794 droplets in total.

An optical microscope (Leica DM 4000M, Diegem, Belgium) with a digital CCD camera (Leica DFC 290 HD) was used to image droplets in four wells, randomly selected from each of the three different droplet generator cartridges. All images were recorded under uniform illumination in a bright field imaging mode, using sufficient magnification (×200). The accuracy of the scale of the images was verified with a calibration grid (Pyser-SGI Ltd, Edenbridge, UK).

The images were analysed by using ImageJ automated image analysis software (v1.47q) following an existing procedure described by Pinheiro et al. [29]. The images were first converted to a bit depth of 8 bits for image processing. The edges of the droplets were identified using the ‘find edges’ algorithm followed by thresholding. To enable detection of the full droplet, a ‘fill holes’ algorithm was run after noise reduction based on a despeckling function. The watershed algorithm implemented in the ImageJ software was used to separate touching droplets. Droplets on the edge of the images were excluded from the analysis. Between 140 and 160 droplets were measured in each of the 12 selected wells providing measurement of 1794 droplets in total.

Several approaches can be followed to measure the 3D droplet volume from the 2D images. One approach is described in detail in reference [29]: the major and minor axes of an elliptical fit to the droplet outline are determined and used to calculate the area (in pixels) of an ellipse of equivalent dimensions. This value was then used to determine the diameter of a circle of an equivalent area, and finally, the volume of a droplet was calculated assuming it has the shape of a perfect sphere. The second approach consists of measuring the average Feret diameter of the droplets as the average of their maximum and minimum Feret diameter. This value can be used as a direct estimate of the diameter of the individual droplets [31].

The length scale of the microscope used at IRMM was calibrated with a calibration grid, leading to an estimated calibration uncertainty of 0.15 %. The National Measurement Institute of Australia (NMIA) produced a more detailed type B uncertainty evaluation of the individual droplet volume measurement via the area equivalent diameter and estimated the relative expanded uncertainty of this measurement as 2.0 %. This uncertainty includes factors such as operator bias in the imaging process, calibration, off-focus image and a component for the (minor) non-sphericity of the droplets.

## Results

### Concentration measurements using dPCR

**Fig. 1:**
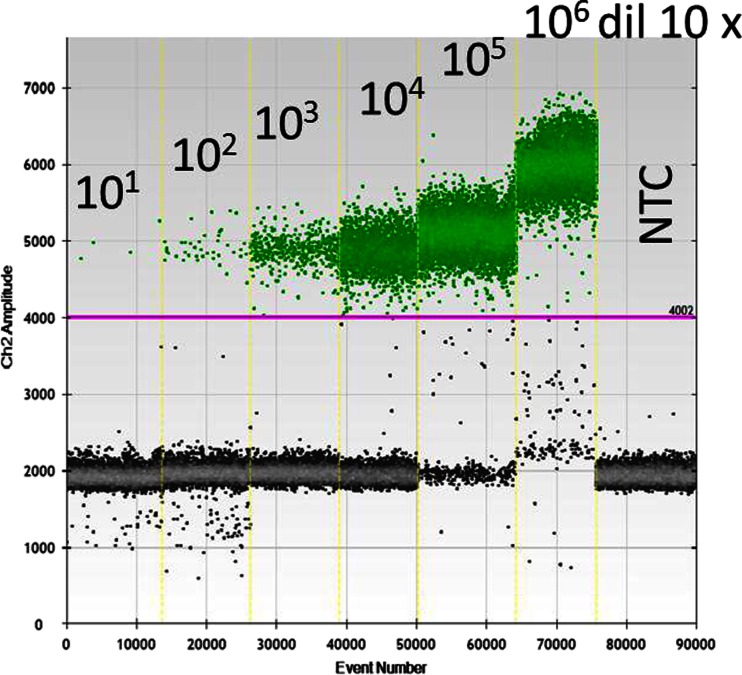
Discrimination of the positive and negative droplets using the *BCR-ABL* ddPCR assay on the ERM-AD623 reference material series. The positive droplets are represented in *green*, whereas the negative droplets are coloured in *grey*

In the first experiment, the comparability of results obtained by two different dPCR platforms (chip vs. droplet based) was investigated by measuring the six plasmid solutions present in the ERM-AD623 set of certified reference materials. A volume of 8.5 μL of each of the six concentrations ranging from 1.08 × 10^6^ to 10 cp/μL was tested in eight replicates in ddPCR. In Fig. 1, the highest concentration was diluted 10× in a 1-mmol/L Tris, 0.01-mmol/L EDTA pH 8.0 buffer to avoid an overloading of positive droplets. The conditions of the PCR assay were optimal as the population of positive droplets could clearly be discriminated from the negative droplets using a threshold value of 4002.

**Fig. 2:**
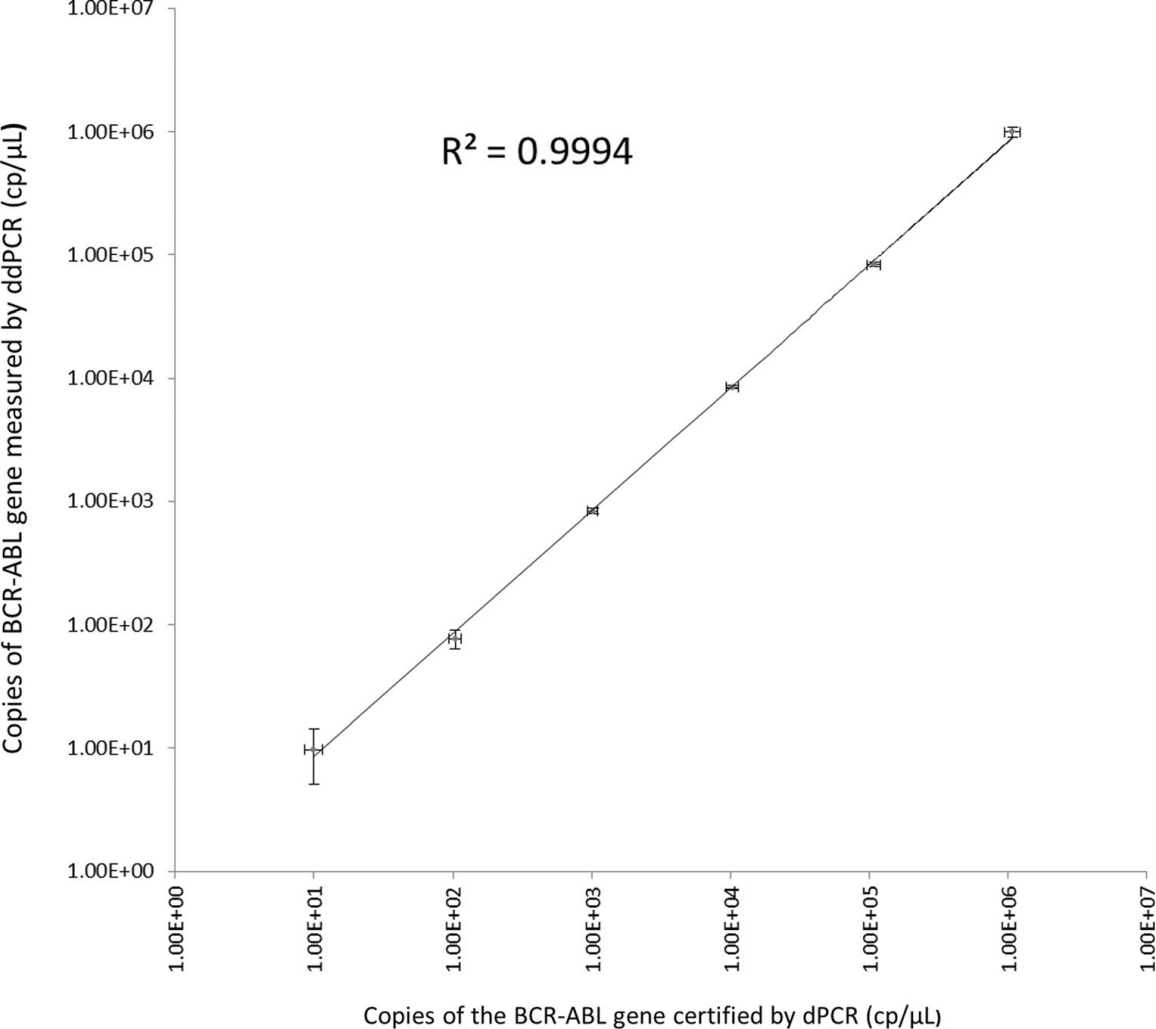
Comparison of both digital PCR platforms using the certified reference material ERM-AD623 as test material. *Horizontal error bars* represent the expanded uncertainty associated to the reference material value assigned by dPCR, whereas the *vertical bars* represent the standard deviation based on eight ddPCR replicates

The certified values as determined by dPCR were plotted against the average copy number concentration determined by ddPCR using the QuantaSoft software version 1.3.2.0 (Fig. 2). A linear regression fitted the data with a coefficient of determination of 0.9994 in a log-log plot suggesting good agreement between the two digital PCR methodologies based on chip partitions and droplets.

The apparently good agreement between the certified values and the measured values was verified by a structured and quantitative approach that allows making a statement on the evidence of any bias. The approach takes into account the certified value and its uncertainty, the measurement result and their respective uncertainties. These uncertainties were subsequently combined, and the expanded uncertainty was compared to the difference between the measured values and the certified values as explained in [32] and detailed in Table 3.

**Table 3:** Comparison between the certified copy number concentrations and concentration estimated by ddPCR in the ERM-AD623 series. A droplet volume of 0.91 nL has been used to calculate the copy number concentration

ERM-AD623	Certified value (cp/μL)	*U*_CRM_	Average measured values (cp/μL), *n* = 8	stdev	*Δ*_m_ (cp/μL)	*u*_m_ (cp/μL)	*U*_*Δ*_ (cp/μL)	Significant bias	% difference
f	10	1.5	9	2	1	1	2	No	12
e	104	10	87	10	17	7	14	Yes	16
d	1020	90	846	80	174	60	121	Yes	17
c	10,300	1000	8851	669	1449	602	1203	Yes	14
b	108,000	11,000	90,951	8105	17,049	6832	13,663	Yes	16
a	1,080,000	130,000	908,543	58,766	171,457	71,333	142,665	Yes	16

The calculated bias is significant with a confidence level of 95 %

*U*_*CRM*_ expanded uncertainty of the certified value; *Δ*_*m*_ absolute difference between mean measured value and certified value; *U*_*Δ*_ combined uncertainty of result and certified value; *u*_*m*_ uncertainty of the measurement result, calculated as the standard deviation divided by the square root of the number of replicates

The copy number concentration of the plasmid measured by ddPCR appeared to be slightly underestimated when compared to the certified values. A statistically significant bias (at 95 % confidence level) varying between 14 and 17 % was observed for ERM-AD623a, ERM-AD623b, ERM-AD623c, ERM-AD623d and ERM-AD623e corresponding to the copy number concentration level ranging from 1,080,000 to 104 cp/μL. No significant bias was observed for the lowest concentration certified at 10 cp/μL.

Underestimation of copy number concentration can have several origins, related either to the PCR assay itself, to the density value used for the gravimetric dilution, to the volume of the assay or to a combination of those three factors. The PCR assays used in this study have been optimised to reduce to a minimum the rain effect, so that the setting of a threshold value to segregate positive droplets from negative droplets was not an issue. Indeed both populations of droplets were clearly segregated (Fig. 1). The plasmid solutions used as test materials were small linearized plasmid to reduce the false negative droplets that may occur with larger unrestricted DNA molecules. Special care was also taken to always dilute the DNA in buffered solutions to reduce potential degradation of the targets prior amplification, reducing as such the probability to miss some droplets containing the target of interest. The PCR platform used was a C1000 Touch Thermo Cycler recommended by Bio-Rad for having uniform, slow ramping temperatures through the plate reducing border plate effects and particularly adapted for this digital PCR application. Special care was also taken to carefully correct the gravimetric dilutions using the density of the pre-mix. Having taken all those precautions, one obvious parameter which is the final volume of the assay had to be investigated.

Therefore, the hypothesis that the observed bias could be the result of an incorrect average droplet volume used in the QuantaSoft software to convert positive droplets into a copy number concentration was tested in subsequent experiments.

### Droplet volume measurements using optical microscopy

To test the above hypothesis, ERM-AD623c, certified to contain 10,300 ± 1000 cp/μL was measured by ddPCR under repeatability and intermediate repeatability conditions by analysing samples from three vials in three independent experiments (*N* = 3) in eight replicates (*n* = 8). The droplets generated in the DG8-138-3008 cartridges were examined under an optical microscope and simultaneously analysed in the droplet reader.

In order to avoid that the examination under the microscope would alter the size of the droplets, the photography of the generated droplets was done in less than 30 min. (The manufacturer recommends starting thermal cycling within 30 min of sealing the PCR plate.)

**Fig. 3:**
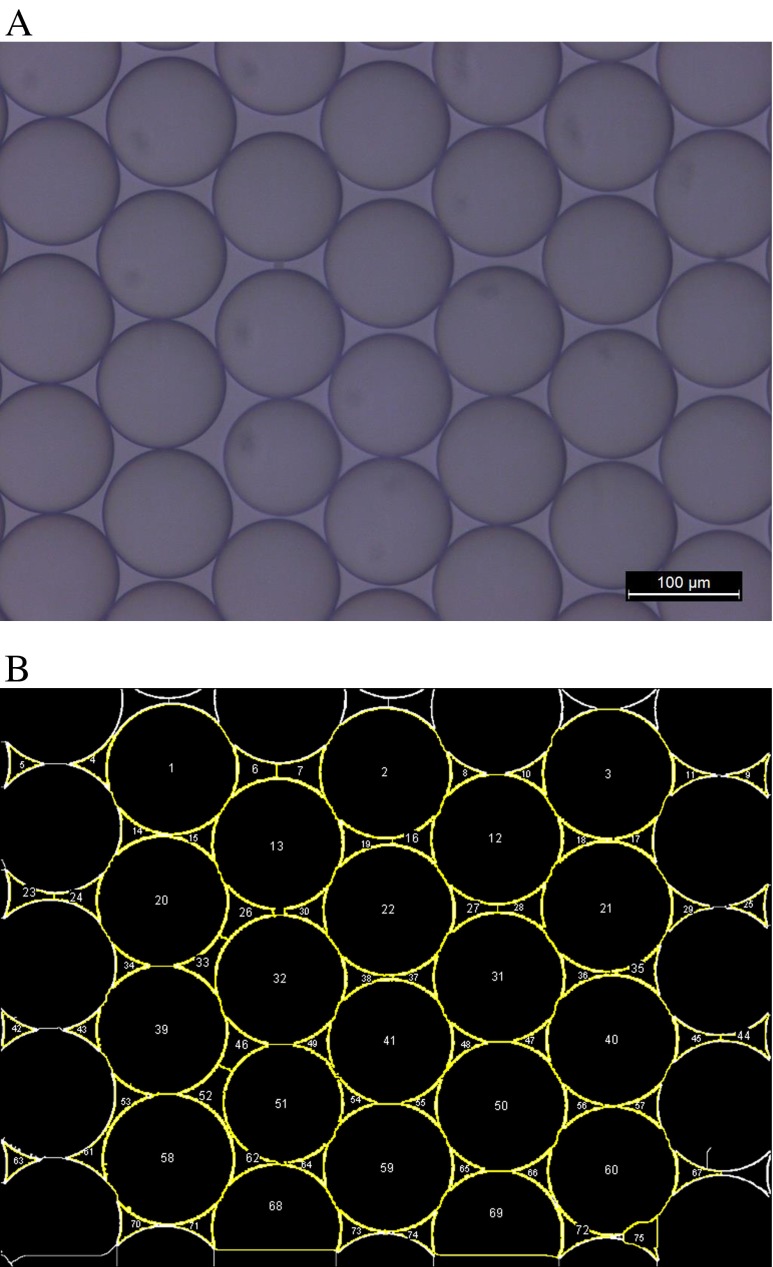
Typical image taken of a monolayer of droplet generated by the droplet generator and observed under an optical microscope (**A**) as well as the image treated by the ImageJ software (v1.47q) to distinguish the droplets from each other and to differentiate the in-between droplet areas from the droplet areas (**B**)

Over the 3 days, a total of 1794 droplets were analysed and imaged by optical microscopy to calculate an average droplet volume. A typical image of a droplet layer is provided in Fig. 3A together with the image obtained after the image analysis in Fig. 3B. For reasons of surface energy minimisation, the liquid droplets in the liquid (oil) matrix have a strong tendency to take a perfectly spherical shape. This shape could be distorted under the action of mechanical stress, for example due to gravity, but during the microscopy investigation, the droplets were submersed in oil, and therefore, the spherical shape is not strongly challenged in the vertical direction. Also, as shown in Fig. 3, there is no indication that the interaction between neighbouring particles results in lateral deformation.

In the 2D image analysis step, two approaches were followed to access the 3D droplet volume (see “Materials and methods” section). The average Feret diameter of the droplets was 3.4 % larger than the diameter calculated using the area equivalent diameter. This observation was explained by noting that the measured Feret diameters are affected by small artificial deviations or tiny protrusions on the droplet perimeter caused by the digital processing of the droplet images. This is the reason why the Feret diameter is consistently larger than the major axis of the best fitting ellipse, and why the area equivalent diameter was chosen to estimate the diameter of the droplets.

The average volume of the droplets generated by the droplet generator in the DG8-186-3008 cartridge (Table 4) was measured as 0.834 nL which is much smaller than the value of 0.91 nL used in the QuantaSoft software version 1.3.2.0 to calculate the copy number concentration.

**Table 4:** Average area equivalent diameters and corresponding volumes of droplets generated by the Bio-Rad Droplet Generator using DG8-186-3001 cartridges. The estimated copy number concentrations of ERM-AD623c in the *BCR-ABL* ddPCR assay were calculated using the corresponding droplet volume that was estimated each day, together with its standard deviation. The uncertainty on the average copy number concentration is an expanded uncertainty with a coverage factor *k* of 2

	Average area equivalent diameter (μm)	Droplet volume (nL)	Average copy number concentration (cp/μL)
Day 1	117.02 ± 1.37 (*n* = 588)	0.839 ± 0.03 (*n* = 588)	9310 ± 196 (*n* = 8)
Day 2	116.59 ± 2.21 (*n* = 605)	0.830 ± 0.04 (*n* = 605)	9586 ± 224 (*n* = 8)
Day 3	116.60 ± 3.18 (*n* = 601)	0.830 ± 0.06 (*n* = 601)	9465 ± 352 (*n* = 8)
Average		0.834	9454 ± 200 (*k* = 2)

Similar experiments have been performed at the NMIA in Sydney reporting volumes of 0.833 and 0.830 nL using ddPCR™ Supermix for Probes and QX200™ dPCR™ EvaGreen® Supermix, respectively (Table 5). To assess the uniformity of the droplet volume within a well, 16 sequential 2 μL samples were collected from a single well of a DG8 cartridge. The relative expanded uncertainty across the entire 16 sub-samples from a single well was 2.4 % (*k* = 2) (Table 5), indicating little variability and good within-well precision. The similarity in values reported from two different laboratories demonstrates good agreement and reproducibility between laboratories applying the same methodology, but using different lots of cartridges and different master mixes.

**Table 5:** Average volume of droplets generated by the Bio-Rad Droplet Generator using either Supermix for Probes or EvaGreen Supermix. The uncertainty on the average copy number concentration is an expanded uncertainty with a coverage factor *k* of 2

Cartridge	Mastermix	Droplet volume (nL)	Total number of droplets
DG8 (production year 2012)^a^	ddPCR™ Supermix for Probes	0.833 ± 0.035	622
DG8 (lot nos. C000021590, C000024241 and C000031616)^a^	QX200™ ddPCR™ EvaGreen® Supermix	0.830 ± 0.027	805
DG8 (production year 2013)^b^	ddPCR™ Supermix for Probes	0.860 ± 0.021	463

^a^Droplets were analysed from three wells from each of five DG8 cartridges (15 wells in total)

^b^Sixteen 2 μL samples were collected sequentially from droplets generated from a single well of a DG8 cartridge and the average droplet volume measured

The relative expanded uncertainty associated to the average droplet volume estimated by IRMM and NMIA for the ddPCR™ Supermix for Probes was 3.6 % (*k* = 2) and 4.2 % (*k* = 2), respectively, indicating an appreciable small variation in the volume of the droplets. For the QX200™ ddPCR™ EvaGreen® Supermix, a very similar relative expanded uncertainty associated to the average droplet volume [3.3 % (*k* = 2)] was estimated indicating also only a small variation of the volume of the droplets with those two different master mixes. On some images, noticeable smaller droplets were observed (results not shown), but according to the manufacturer, those very small droplets are eliminated by the reader and are not used by the software as countable droplets.

## Discussion

**Fig. 4:**
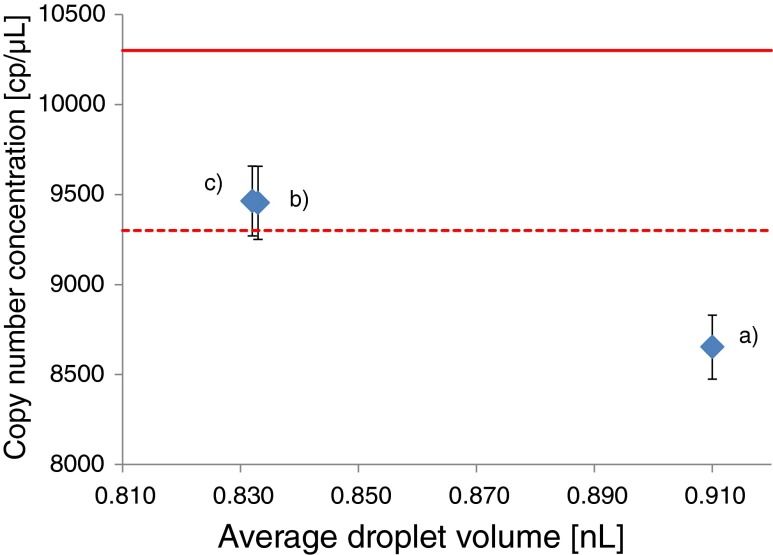
Influence of the value used for average droplet volume in Eq. (1) on the copy number concentration (displayed with their standard deviation) determined by ddPCR. (**a**) With using a volume of 0.91 nL used in the QuantaSoft version 1.3.2.0, (**b**) with using an average volume of 0.834 nL measured in this study and (**c**) with using an average volume of 0.833 nL measured by NMIA. The *continuous line* represents the certified value assigned by dPCR and the *dotted line* displays the 95 % lower confidence interval

The droplet volume measured using the DG8 cartridge is slightly smaller than the volume generated using the beta-version of the droplet generator [29] demonstrating the importance of re-measuring the droplet volume when new generators or cartridges are released. Equation (1) illustrates that the copy number concentration is inversely related to the average droplet volume (*V*_d_). Hence, if the number of positive droplets (*H*) and total number of droplets (*C*) are constant, the use of a smaller droplet volume (*V*_d_) in the formula will increase the estimated copy number concentration. This is illustrated in Fig. 4, showing how the estimated copy number concentration per microliter of the plasmid in ERM-AD623c varies depending on the droplet volume (*V*_d_) used in the equation.

The copy number concentration measured by ddPCR for the ERM-AD623c was 9454 cp/μL with an expanded uncertainty of 200 cp/μL taking into consideration the measured droplet volume of 0.834 nL in our experimental setup (Table 4). The uncertainty associated to the average copy number concentration was determined taking into account the variance of the repeatability estimated by the residual of the ANOVA and the variance of the between-day repeatability or the intermediate precision. The certified concentration of 10,300 cp/μL remains slightly higher than the value measured by ddPCR. However, the remaining bias of 8 % is statistically no longer significant as it is smaller than the combined uncertainty of the certified value and the measured value.

The bias observed in the first set of experiments using the manufacture-specified droplet volume of 0.91 nL (Fig. 4(a)) disappears when taking into account the real droplet volume as determined in this study (Fig. 4(c)). Indeed, the measured copy number concentration taking into account the real droplet volume increased by 8 % bringing it closer to the certified values assigned by dPCR. When applying the measured volume of 0.834 nL to recalculate the copy number concentrations reported in Table 3, the bias observed for the values on the ERM-AD623 series is not statistically significant anymore. The copy number concentrations determined by ddPCR remain nevertheless slightly lower (between 4 and 9 %) than those reported by dPCR. This could be attributed to a variability in the partition volume in the Fluidigm 12.765 digital array, since the relative standard uncertainty of the average partition volume for the 12.765 digital array has previously been estimated at 5 % [27]. More extensive studies to verify the accuracy of the partition volume in dPCR would be required to exclude this possibility.

The finding of two independent laboratories concerning the droplet volume generated by the DG8-186-3008 has been communicated to the instrument producer Bio-Rad, who has since launched a newer version of the QuantaSoft software (version 1.6.6) using an average droplet volume of 0.85 nL. This newer software version coincides with the placing on the market of a newer droplet generator cartridge (DG8-186-4008) that is supposed to replace the earlier cartridges [Bio-Rad, personal communication]. The value of 0.85 nL used in the software version 1.6.6 is not accompanied with an uncertainty but is lowered enough to avoid the significant bias observed with the earlier software version. No further details on the method used to determine this volume could be obtained from Bio-Rad.

**Fig. 5:**
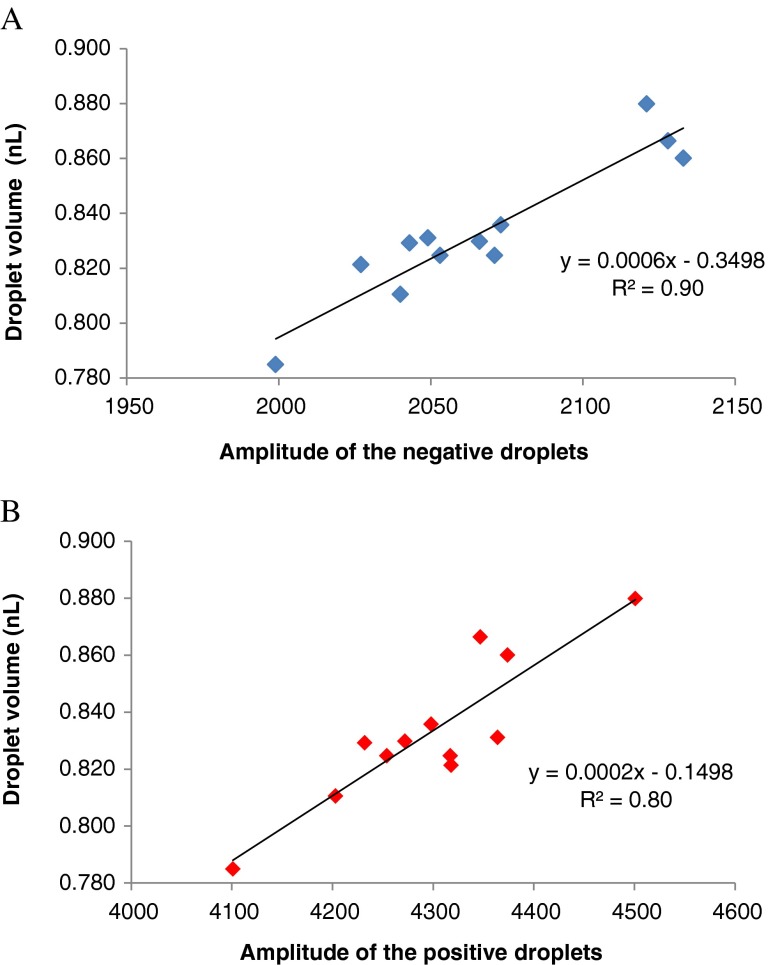
Relationship between the average amplitude of the fluorescence in negative (**A**) and positive (**B**) droplets in dependence on the droplet size. *Each diamond* represents the average droplet volume and the average fluorescence amplitude of droplets generated from a single well in a droplet generator cartridge

The variability in the volume of droplets generated from different cartridge wells and across different channels correlated with changes in the average fluorescence amplitude reported by the QuantaSoft software (Fig. 5A, B). A slightly stronger fluorescence signal for both positive and negative droplets was observed in larger droplets. This is not unexpected since larger droplets will contain more fluorescence molecules than smaller droplets. It means that a small variation of the fluorescence amplitude among replicates of the same assay (around 10 % in this study) is an indication that droplets with different average volumes were produced in each channel of the droplet generator. In an optimised assay, it is theoretically possible to recalculate the copy number concentration with a correction factor that takes into account the variability of the droplet volume among replicates. This feature may be exploited in the future to improve the precision of ddPCR measurements.

## Conclusion

In this study, a reference material certified for the copy number concentration of a plasmid solution over a concentration range from 1 million to 10 cp/μL was used to compare the certified values determined by dPCR with results from another digital PCR method based on droplet emulsion. Copy number concentrations measured by those two digital PCR methods were in good agreement, if the real average volume of the droplets was used to recalculate the assay volume. The volume of 0.91 nL used in the QuantaSoft software version 1.3.2.0 was found to be inaccurate and was the source of the bias observed between the results using the two digital PCR platforms. This study illustrates on the benefit of using a certified reference material to validate methods and procedures involving digital PCR and the risk of relying on data generated by a software which makes assumptions on the value of one or more experimental variables.

For many clinical applications, where copy numbers are expressed as ratios, the uncertainty on the volume—provided the volume stays constant—is not so critical. However, when digital PCR is used to assign ‘absolute’ copy number concentrations, the volume of the assay needs ideally to be confirmed. The droplet volume of 0.85 nL used in the latest software version from Bio-Rad is a value that is very close to the average droplet volume measured in this paper. Assuming that other parameters that affect the number of positive droplets, the total number of droplets and the dilution process are under control, an ‘absolute’ quantification of copy number using that pre-set volume can be reliable. However, digital PCR-based enumeration techniques to quantify bacteria, viruses or whole cells that are encapsulated in the droplets from which the volume is unknown or fluctuating with the applied experimental conditions will require the real volume of the droplets to be taken into consideration [33, 34].

## References

[1] Raso A, Biassoni R (2014). Twenty years of qPCR: a mature technology?. Methods Mol Biol.

[2] VanGuilder HD, Vrana KE, Freeman WM (2008). Twenty-five years of quantitative PCR for gene expression analysis. Biotechniques.

[3] Klatte M, Bauer P (2009). Accurate real-time reverse transcription quantitative PCR. Methods Mol Biol.

[4] Lee SB, McCord B, Buel E (2014). Advances in forensic DNA quantification: a review. Electrophoresis.

[5] Horsman KM, Bienvenue JM, Blasier KR, Landers JP (2007). Forensic DNA analysis on microfluidic devices: a review. J Forensic Sci.

[6] Hall Sedlak R, Jerome KR (2014). The potential advantages of digital PCR for clinical virology diagnostics. Expert Rev Mol Diagn.

[7] Sedlak RH, Jerome KR (2013). Viral diagnostics in the era of digital polymerase chain reaction. Diagn Microbiol Infect Dis.

[8] Kumar A, Rahal A, Chakraborty S, Verma AK, Dhama K (2014). Mycoplasma agalactiae, an etiological agent of contagious agalactia in small ruminants: a review. Vet Med Int.

[9] Beer (1852). Bestimmung der Absorption des rothen Lichts in farbigen Flüssigkeiten. Ann Phys Chem.

[10] Singer VL, Jones LJ, Yue ST, Haugland RP (1997). Characterization of PicoGreen reagent and development of a fluorescence-based solution assay for double-stranded DNA quantitation. Anal Biochem.

[11] Bowers RM, Dhar AK (2011). Effect of template on generating a standard curve for absolute quantification of an RNA virus by real-time reverse transcriptase-polymerase chain reaction. Mol Cell Probes.

[12] Chen J, Kadlubar FF, Chen JZ (2007). DNA supercoiling suppresses real-time PCR: a new approach to the quantification of mitochondrial DNA damage and repair. Nucleic Acids Res.

[13] Hou Y, Zhang H, Miranda L, Lin S (2010). Serious overestimation in quantitative PCR by circular (supercoiled) plasmid standard: microalgal pcna as the model gene. PLoS One.

[14] Wu YL, Savelli SL, Yang Y, Zhou B, Rovin BH, Birmingham DJ, Nagaraja HN, Hebert LA, Yu CY (2007). Sensitive and specific real-time polymerase chain reaction assays to accurately determine copy number variations (CNVs) of human complement C4A, C4B, C4-long, C4-short, and RCCX modules: elucidation of C4 CNVs in 50 consanguineous subjects with defined HLA genotypes. J Immunol.

[15] Øvstebø R, Haug KB, Lande K, Kierulf P (2003). PCR-based calibration curves for studies of quantitative gene expression in human monocytes: development and evaluation. Clin Chem.

[16] Toyota A, Akiyama H, Sugimura M, Watanabe T, Sakata K, Shiramasa Y, Kitta K, Hino A, Esaka M, Maitani T (2006). Rapid quantification methods for genetically modified maize contents using genomic DNAs pretreated by sonication and restriction endonuclease digestion for a capillary-type real-time PCR system with a plasmid reference standard. Biosci Biotechnol Biochem.

[17] Yukl SA, Kaiser P, Kim P, Li P, Wong JK (2014). Advantages of using the QIAshredder instead of restriction digestion to prepare DNA for droplet digital PCR. Biotechniques.

[18] Sivaganesan M, Seifring S, Varma M, Haugland RA, Shanks OC (2008). A Bayesian method for calculating real-time quantitative PCR calibration curves using absolute plasmid DNA standards. BMC Bioinforma.

[19] Burns M, Corbisier P, Wiseman G, Valdivia H, McDonald P, Bowler P, Ohara K, Schimmel H, Charels D, Damant A, Harris N (2006). Comparison of plasmid and genomic DNA calibrants for the quantification of genetically modified ingredients. Eur Food Res Technol.

[20] Caprioara-Buda M, Meyer W, Jeynov B, Corbisier P, Trapmann S, Emons H (2012). Evaluation of plasmid and genomic DNA calibrants used for the quantification of genetically modified organisms. Anal Bioanal Chem.

[21] Corbisier P, Vincent S, Schimmel H, Kortekaas AM, Trapmann S, Burns M, Bushell C, Akgoz M, Akyürek S, Dong L, Fu B, Zhang L, Wang J, Pérez Urquiza M, Bautista JL, Garibay A, Fuller B, Baoutina A, Partis L, Emslie K, Holden M, Chum WY, Kim HH, Phunbua N, Milavec M, Zel J, Vonsky M, Konopelko LA, Lau TLT, Yang B, Hui MHK, Yu ACH, Viroonudomphol D, Prawettongsopon C, Wiangnon K, Takabatake R, Kitta K, Kawaharasaki M, Parkes H (2012) CCQM-K86/P113.1: relative quantification of genomic DNA fragments extracted from a biological tissue. Metrologica 1A (Technical Supplement) vol 49

[22] Vogelstein B, Kinzler KW (1999). Digital PCR. Proc Natl Acad Sci U S A.

[23] Haynes RJ, Kline MC, Toman B, Scott C, Wallace P, Butler JM, Holden MJ (2013). Standard reference material 2366 for measurement of human cytomegalovirus DNA. J Mol Diagn.

[24] Deprez L, Mazoua S, Corbisier P, Trapmann S, Schimmel H, White H, Emons H (2012) The certification of the copy number concentration of solutions of plasmid DNA containing a BCR–ABL b3a2 transcript fragment. Certified reference material: ERM-AD623a, ERM-AD623b, ERM-AD623c, ERM-AD623d, ERM-AD623e, ERM-AD623f. Luxembourg: Publications Office of the European Union, 2012; Report number EUR 25248; ISBN 978-92-79-23343-2

[25] Sanders R, Mason DJ, Foy CA, Huggett JF (2013). Evaluation of digital PCR for absolute RNA quantification. PLoS One.

[26] Dong L, Meng Y, Wang J, Liu Y (2014). Evaluation of droplet digital PCR for characterizing plasmid reference material used for quantifying ammonia oxidizers and denitrifiers. Anal Bioanal Chem.

[27] Bhat S, Herrmann J, Armishaw P, Corbisier P, Emslie KR (2009). Single molecule detection in nanofluidic digital array enables accurate measurement of DNA copy number. Anal Bioanal Chem.

[28] Huggett JF, Foy CA, Benes V, Emslie K, Garson JA, Haynes R, Hellemans J, Kubista M, Mueller RD, Nolan T, Pfaffl MW, Shipley GL, Vandesompele J, Wittwer CT, Bustin SA (2013). Guidelines for minimum information for publication of quantitative digital PCR experiments. Clin Chem.

[29] Pinheiro LB, Coleman VA, Hindson CM, Herrmann J, Hindson BJ, Bhat S, Emslie KR (2012). Evaluation of a droplet digital polymerase chain reaction format for DNA copy number quantification. Anal Chem.

[30] White H, Deprez L, Corbisier P, Hall V, Lin F, Mazoua S, Trapmann S, Aggerholm A, Andrikovics H, Akiki S, Barbany G, Boeckx N, Bench A, Catherwood M, Cayuela JM, Chudleigh S, Clench T, Colomer D, Daraio F, Dulucq S, Farrugia J, Fletcher L, Foroni L, Ganderton R, Gerrard G, Gineikienė E, Hayette S, El Housni H, Izzo B, Jansson M, Johnels P, Jurcek T, Kairisto V, Kizilors A, Kim DW, Lange T, Lion T, Polakova KM, Martinelli G, McCarron S, Merle PA, Milner B, Mitterbauer-Hohendanner G, Nagar M, Nickless G, Nomdedéu J, Nymoen DA, Leibundgut EO, Ozbek U, Pajič T, Pfeifer H, Preudhomme C, Raudsepp K, Romeo G, Sacha T, Talmaci R, Touloumenidou T, Van der Velden VH, Waits P, Wang L, Wilkinson E, Wilson G, Wren D, Zadro R, Ziermann J, Zoi K, Müller MC, Hochhaus A, Schimmel H, Cross NC, Emons H (2014). A certified plasmid reference material for the standardisation of BCR-ABL1 mRNA quantification by real-time quantitative PCR. Leukemia.

[31] ISO 13322-1 (2004) Particle size analysis—image analysis methods—part 1: static image analysis methods

[32] Linsinger T (2010) ERM Application note 1: comparison of a measurement result with the certified value https://ec.europa.eu/jrc/sites/default/files/erm_application_note_1_en.pdf. Accessed 11 Nov 2014

[33] Zeng Y, Novak R, Shuga J, Smith MT, Mathies RA (2010). High-performance single cell genetic analysis using microfluidic emulsion generator arrays. Anal Chem.

[34] Dreo T, Pirc M, Ramšak Ž, Pavšič J, Milavec M, Zel J, Gruden K (2014). Optimising droplet digital PCR analysis approaches for detection and quantification of bacteria: a case study of fire blight and potato brown rot. Anal Bioanal Chem.

